# Built Environment and HIV Linkage to Care in Rural South Africa

**DOI:** 10.1177/0272684X211006590

**Published:** 2021-04-04

**Authors:** Nosipho Shangase, Brian Pence, Sheri A. Lippman, Mi-Suk Kang Dufour, Chodziwadziwa Whiteson Kabudula, F. Xavier Gómez-Olivé, Kathleen Kahn, Audrey Pettifor

**Affiliations:** 1 Department of Epidemiology, Gillings School of Global Public Health, University of North Carolina, Chapel Hill, North Carolina, United States; 2 Department of Medicine, University of California, San Francisco, California, United States; 3 MRC/Wits Rural Public Health and Health Transitions Research Unit (Agincourt), School of Public Health, Faculty of Health Sciences, University of the Witwatersrand, Johannesburg, South Africa

**Keywords:** HIV linkage to care, Agincourt Health and Demographic Surveillance System, built environment, universal test and treat, South Africa

## Abstract

**Background:**

We assessed built environment (residential density, landuse mix and aesthetics) and HIV linkage to care (LTC) among 1,681 (18–49 years-old) residents of 15 Mpumalanga villages, South Africa.

**Methods:**

Multilevel models (linear-binomial) were used for the association between built environment, measured using NEWS for Africa, and LTC from a clinical database of 9 facilities (2015–2018). Additionally, we assessed effect-measure modification by universal test-and-treat policy (UTT).

**Results:**

We observed, a significant association in the adjusted 3-month probability of LTC for residential density (risk difference (RD)%: 5.6, 95%CI: 1.2–10.1), however, no association for land-use mix (RD%: 2.4, 95%CI: −0.4, 5.2) and aesthetics (RD%: −1.2, 95%CI: −4.5–2.2). Among those diagnosed after UTT, residents of high land-use villages were more likely to link-to-care than those of low land-use villages at 12 months (RD%: 4.6, 95%CI: 1.1–8.1, p < 0.04), however, not at 3 months (RD%: 3.0, 95%CI: −2.1–8.0, p > 0.10).

**Conclusion:**

Findings suggest, better built environment conditions (adequate infrastructure, proximity to services etc.) help facilitate LTC. Moreover, UTT appears to have a protective effect on LTC.

Worldwide, South Africa (SA) has the largest HIV treatment program^1^ with approximately 3.7 million people on treatment.^2^ Despite the scale up of HIV treatment through the universal test and treat (UTT) policy, only around 56% of adults diagnosed with HIV are on antiretroviral therapy (ART),^2,3^ and the number of new infections remains high.^4^ HIV linkage to care (LTC) ‒ which is the initial utilization of HIV-related healthcare services such as treatment and counseling services ‒ is an entry point into the HIV care continuum.^5,6^ Timely linkage to care and immediate initiation of ART leads to optimal clinical outcomes such as HIV viral suppresion^5,7–10^ – a key factor to treatment-as-prevention. Thus, strengthening early HIV LTC is necessary for improving treatment and prevention efforts.^3^

Most research on HIV linkage to care has focused on individual-level determinants^10–15^ with a shortage of studies focusing on structural factors such as the built environment ‒ which refers to spaces in which people live and work such as homes, schools and recreational areas.^16^ Built environment has been shown to have an impact on health outcomes, consequently, public health research on this association has increased since the early 2000s.^17–20^ Although there have been studies looking at the effects of built environment on sexual risk behavior,^21^ risk of HIV infection,^22^ and on HIV treatment adherence,^23^ a gap remains in understanding the influence of neighborhood-level factors on HIV linkage to care.^18^ Understanding this association is crucial for South Africa because there are still communities lacking basic built environment infrastructure such as clean water and adequate housing, and residents of rural communities still encounter barriers in accessing health care.^24^

One mechanism supported by research is that built environment affects HIV linkage to care through influencing health behaviors.^21,25–27^ The neighborhood infrastructure such as road conditions, and resources in the neighborhood can create opportunities for residents to seek care. For instance, availability and close proximity of HIV testing services can promote health-seeking behaviors like HIV testing.^26,27^ Presence of these conditions enable residents to utilize healthcare facilities and ultimately affects whether residents link to care or not. However, it is important to note, HIV stigma and discrimination could prevent residents from using nearby HIV health services.^28^ Using cross-sectional data, we examined the relationship between built environment and HIV linkage to care among residents of the MRC/Wits-Agincourt study area in rural Mpumalanga, SA. In addition, we assessed effect measure modification of this association by implementation of the UTT policy.

## Methods

### Study Design and Study Setting

**Figure 1.**
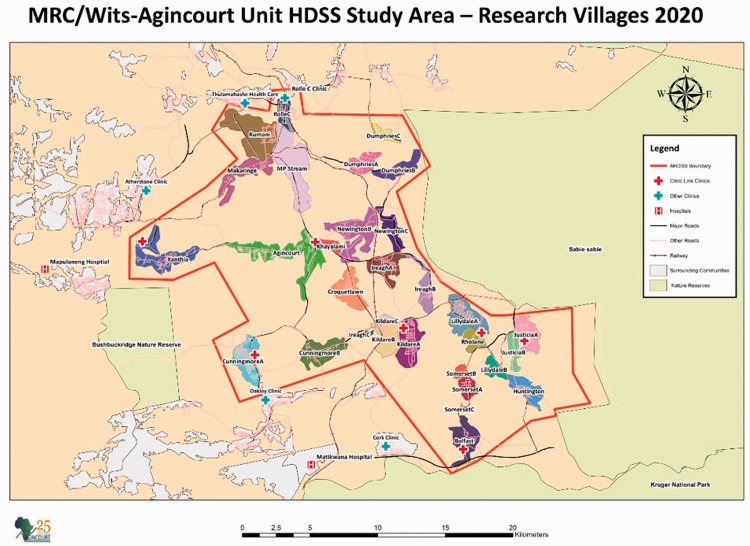
MRC/Wits-Agincourt Unit HDSS Study (Research Villages). Source: https://www.agincourt.co.za/?page_id=1896.

This cross-sectional study utilized longitudinal clinic-based data nested within the Tsima cluster randomized trial which took place from August 1, 2015 to August 1, 2018. The parent study is a community mobilization intervention that was conducted in 15 villages (8 intervention and 7 control) aimed at improving engagement in HIV testing and care.^29^ The study site is located in a sub-district of Bushbuckridge in the rural northeast of Mpumalanga Province near the Mozambique border, and covered by the Agincourt Health and Socio-Demographic Surveillance System (AHDSS), which is run by the Medical Research Council/Wits University Rural Public Health and Health Transitions Research Unit (see Figure 1).^30^ The AHDSS is part of a the former Gazankulu “homeland,^30^ and it covers 31 rural villages with a population of around 116,000.^29^ The study area has infrastructure problems including gravel roads, limited access to electricity and water, poor sanitation, high unemployment and relatively poor quality education.^31^ Agincourt experiences high labor migration to urban areas with 19% and 31% female and male migrants, respectively.^31,32^ Due to dry climate, agriculture is not the main form of local employment,^31^ however households supplement purchased food with home-grown crops.^33^ In this area, the main form of transportation is minibus-taxis.^32^ The sub-district has 9 public clinics, including 2 health centers, as well as 3 district hospitals between 25 and 60 kms from the study area.^31^ Health facilities in the Agincourt sub-district have shown deficiencies in meeting minimum service delivery demands.^34^

### Study Population

The study cohort includes participants: 1) between ages 18 to 49 years, 2) who tested HIV positive at one of the 9 AHDSS health facilities, and 3) who had no indication of HIV diagnosis or HIV-related care prior to 1 August 2015. We excluded individuals who were diagnosed with HIV or who had a record indicating that they received HIV-related care such as ART treatment, CD4 count or viral load testing prior to 1 August 2015 because LTC data was not captured prior to the Tsima study. Our analysis is limited to residents of the 15 villages in the main study.

### Measures

#### Built Environment

To assess built environment, we randomly selected global positioning system (GPS) coordinates (n = 4 per village) in the 15 villages using ArcGIS software (ESRI 2016. ArcGIS Desktop: Release 10.4.1. Redlands, CA: Environmental Systems Research Institute). Three trained individuals collected data at the randomly selected points within a radius of approximately 0.5 miles (∼1 km). We assessed interrater reliability using intraclass correlation coefficients (ICC) for residential density, land-use mix and aesthetics to ensure homogeneity between raters, and re-trained if there were variations between the raters. Overall, the ICC was low for residential density (0.20), indicating disagreement between the raters, and relatively high for aesthetics and land-use mix, indicating raters were in agreement (Table 1).

**Table 1.** Descriptive Statistics for the Built Environment Measures (N = 15 Villages).

Variable	Range	Mean (SD)	Interrater reliability (ICC)^†^
Residential density	177.5–219.17	193.91 (14.56)	0.20
Land-use mix	2.78–4.22	3.47 (0.42)	0.88
Aesthetics	2.47–3.08	2.66 (0.17)	0.55

^†^ICC closer to 1 indicates little variation between the raters.

**Figure 2.**
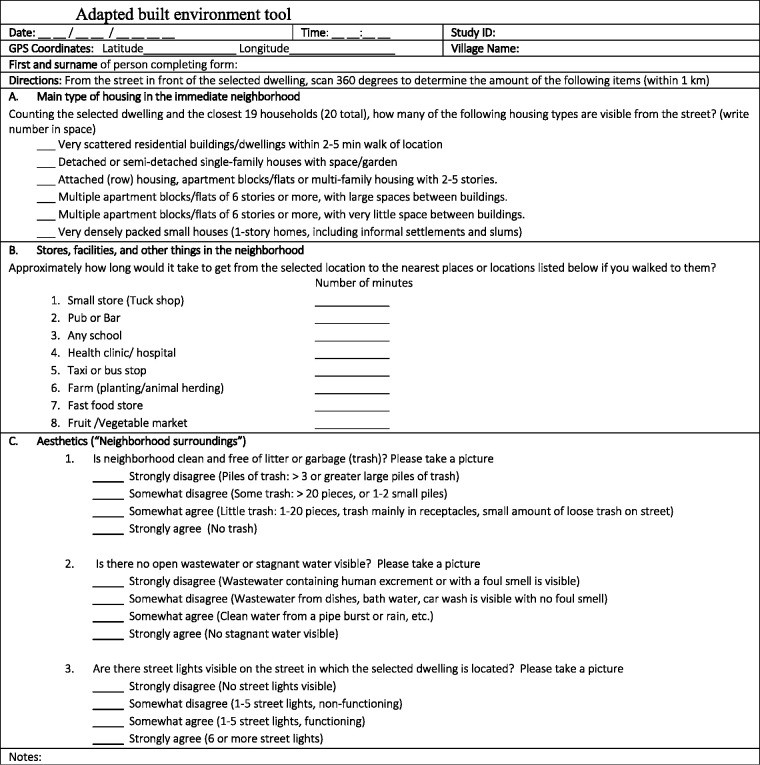
Adapted Built Environment Tool.

The built environment was measured using items from the Neighborhood Environment Walkability Scale for Africa (NEWs)^35^ (see Figure 2, below). Items were scored as suggested in NEWS^35^ – with a higher score representing higher walkability for all components, which is considered a positive attribute. Features assessed were: 1) residential density (six items), 2) land-use mix (eight items), and 3) aesthetics (three items). Residential density is a measure of residential dwellings per unit area of land.^36,37^ The tool captured the frequency of different types of dwellings observed, which was then translated into: None (1), A few (2), some (3), most (4), and all (5). Then, the items were weighted relative to the density of single-family detached home (for example, “very densely packed small houses” were considered to be 75 times more dense than a single family home) and were summed to create a residential density score.^35^ Land-use mix measures proximity to non-residential services or resources such as school and clinics.^37^ Responses were scored as 5 for destinations within 5 minutes of walking and as 1 for destinations more than 30 minutes. Aesthetics were characterized using questions focused on the appearance of the neighborhood, such as, “is there no open wastewater or stagnant water visible?” Responses were scored as 1 for strongly disagree and 4 for strongly agree. Scores were consolidated and a mean score was calculated separately for land-use mix and for aesthetics items (see Table 1). Higher built environment was classified as 1 (binary) if the score was equal or above the mean for each of the items on the scale.

#### HIV Linkage to Care

The parent study used a health facility-based tracking system to measure HIV linkage to care (clinic link). Individuals were classified as linked to care at two time points ‒ 3 months and also at 12 months following diagnosis ‒ if they met one of the 3 criteria: 1) CD4 test results delivery date within 3 or 12 months after first diagnosis date, 2) follow up visit with an indication of HIV treatment or care within 3 or 12 months, or 3) CD4 or viral load test within 3 or 12 months after the first diagnosis date. Loss to care was defined as lack of linkage within 12 months of first diagnosis.

### Statistical Analysis

We used individuals as the unit of analysis ‒ the village built environment scores (residential density, land-use, and aesthetics) were assigned to each individual based on their census village of residence that corresponds with year of diagnosis. We fit multilevel linear binomial regression models with a random intercept for villages with 95% confidence intervals (CI). For continuous variables, functional form was assessed by comparing linear to flexible models (cubic spline) using the likelihood ratio test (LRT) and the Akaike information criterion (AIC). Models were adjusted for absolute socio-economic status (SES), which is a measure of household assets,^38^ and for the intervention arm. Other covariates included: tuberculosis (presence of TB diagnosis date or TB treatment date was coded as 1, binary); health facility type (0 = community health center, 1= clinic); pregnancy was coded as binary (1 = “yes”), and patients diagnosed with HIV before 1 September 2016 were classified as 1 for prior to the UTT policy change (binary). In addition, we assessed for effect measure modification by UTT using the Wald chi-square test with *p*-value <0.10 considered as presence of effect measure modification. Statistical analyses were performed using SAS, version 9.4 (SAS Institute, Inc., Cary, North Carolina). IRB approval for this analysis was obtained from the University of North Carolina at Chapel Hill (UNC) and the University of the Witwatersrand (Wits) Human Research Ethics Committee; approval for the parent study was obtained from University of California San Francisco, UNC and Wits. All clinical visit data are de-identified at the research site prior to analytic work.

## Results

### Sample Description

Our analysis included a sample of 1,681 participants with a mean (SD) age of 31.4 (7.97) years (Table 2). Approximately 72.0% of participants were female, and 34.5% of the female participants were pregnant at the time of diagnosis. Around 63.9% of participants were diagnosed with TB or were treated for TB. The majority of participants attended community health centers (53.0%). Among all participants, 44.4% were diagnosed before the universal test and treat policy was implemented. Approximately 55.7%, 55.3% and 40.3% of the participants were from villages with high residential density, land-use mix and aesthetics, respectively.

**Table 2.** Demographic and Clinical Characteristics of 1,681 Eligible Residents of the AHDSS in 2015–2018.

	Total		Probability of LTC within 3 months of diagnosis		Probability of LTC within 12 months of diagnosis*	
	N	Column %	n	Row %	n	Row %
						
Overall	1,681		1,486	88.4	1,540	91.6
Age, mean (SD)	31.4 (7.97)		31.6 (7.99)		31.6 (7.96)	
Sex						
Female	1,210	72.0	1,066	88.1	1,107	91.5
Male	471	28.0	420	89.2	433	91.9
CD4 count (cell/mm^3^)						
< 200	531	38.0	503	37.8	515	37.5
200–349	391	28.0	369	27.7	385	28.1
350–500	253	18.1	245	18.4	251	18.3
> 500	224	16.0	213	16.0	221	16.1
Missing	282					
Pregnant ^†^						
Yes	417	34.5	387	92.8	401	96.2
No	793	65.5	679	85.6	706	89.0
TB						
Yes	1,074	63.9	1,008	93.9	1,047	97.5
No	607	36.1	478	78.8	493	81.2
Health facility						
Health centre	890	53.0	780	87.6	808	90.8
Clinic	790	47.0	705	89.2	731	92.5
Missing	1					
Diagnosed before the universal test and treat policy					
Yes	747	44.4	675	90.4	706	94.5
No	934	55.6	811	86.8	834	89.3
Residential density						
High	937	55.7	833	88.9	861	91.9
Low	744	44.3	653	87.8	679	91.3
Land-use						
High	929	55.3	830	89.3	859	92.5
Low	752	44.7	656	87.2	681	90.6
Aesthetics						
High	677	40.3	595	87.9	618	91.3
Low	1,004	59.3	891	88.8	922	91.8

^†^Includes only female participants.

*Consists of participants who linked to care within 12 months of HIV diagnosis, including those who linked to care within 3 months of diagnosis.

### HIV Linkage to Care

Overall, 88.4% of participants linked to care within 3 months of HIV diagnosis. Among pregnant females, 88.1% linked to care within 3 months of diagnosis. Also, 93.9% of participants diagnosed or treated for tuberculosis linked to care within 3 months of diagnosis. The proportion of clinic attendees who linked within 3 months was higher (89.2%) compared to community health centre attendees who linked within 3 months (87.6%). Approximately, 86.8% of those who were diagnosed after the UTT policy linked to care within 3 months compared to 90.4% of those who were diagnosed before the policy.

For high residential density, the probability of HIV linkage to care was 88.9% at 3 months. As illustrated in Table 3, there was a difference in the adjusted 3-month risk of HIV linkage to care for residents with high residential density compared to those with low residential density (aRD% = 5.6, 95% CI: 1.2% to 10.1%), and also at 12 months of HIV linkage to care (aRD%: 3.2, 95% CI: 1.1% to 5.4%). With respect to land-use mix, the proportion of linkage among participants with high land-use mix was 89.3% at 3 months. The 3-month effect of land-use mix was similar between those with high vs. low land-use mix (aRD% =2.4, 95% CI: -0.4% to 5.2%), and at 12 months. For aesthetics, 87.9% of participants with high aesthetics linked to care within 3 months of HIV diagnosis. The 3-month adjusted risk of HIV LTC was similar among participants with high aesthetics compared to those who live in villages with low aesthetics (aRD%: -1.2, 95% CI: -4.5% to 2.2%) and also, at 12 months.

**Table 3.** Crude and Adjusted Multilevel Regression Analysis for the Effect of Residential Density, Land Use, Aesthetics on 3-Month and 12-Month Risk of HIV LTC.

	LTC within 3 months of diagnosis		LTC within 12 months of diagnosis	
	Crude	Adjusted^‡^	Crude	Adjusted^‡^
	RD% (95% CI)	RD% (95% CI)	RD% (95% CI)	RD% (95% CI)
Residential density				
Low	0	0	0	0
High	1.2 (−2.3 to 4.8)	5.6 (1.2 to 10.1)	0.7 (−2.0 to 3.5)	3.2 (1.1 to 5.4)
Land-use				
Low	0	0	0	0
High	2.1 (−0.7 to 5.0)	2.4 (−0.4 to 5.2)	2.0 (−0.6 to 4.5)	2.2 (−0.2 to 4.5)
Aesthetics				
Low	0	0	0	0
High	−0.9 (−5.0 to 3.1)	−1.2 (−4.5 to 2.2)	−0.6 (−3.4 to 2.3)	−0.8 (−3.8 to 2.2)

When stratified by the universal test and treat policy (Table 4), linkage within 12 months was higher among those from villages with high land-use mix than those from villages with low land-use mix (aRD%: 4.6, 95% CI: 1.1% to 8.1%, LRT = 4.27, *p* = 0.0387) after the policy was implemented, but not at 3 months of diagnosis (aRD%= 3.0, 95% CI: -2.1% to 8.0%). Furthermore, there was no indication of presence of effect measure modification (p-value > 0.10) for the resident density and aesthetics both at 3 and 12 months.

**Table 4.** Adjusted^‡^ Measure of Built Environment and HIV Linkage to Care Association Within Levels of Universal Test and Treat.

	LTC within 3 months of diagnosis			LTC within 12 months of diagnosis		
	Prior UTT	UTT	Wald X^2^	Prior UTT	UTT	Wald X^2^
	RD% (95% CI)	RD% (95% CI)	(*p*-value)	RD% (95% CI)	RD% (95% CI)	(*p*-value)
Residential density	6.2 (−2.7 to 15.0)	7.0 (0.2 to 13.8)	0.04 (0.8475)	4.0 (−2.9 to 11.0)	4.9 (0.2 to 9.7)	0.06 (0.8109)
Land-use	1.6 (−3.0 to 6.3)	3.0 (−2.1 to 8.0)	0.1 (0.7473)	−1.1 (−4.9 to 2.8)	4.6 (1.1 to 8.1)	4.27 (0.0387)
Aesthetics	0.1 (−3.5 to 3.7)	−2.0 (−8.2 to 4.2)	0.26 (0.6122)	−0.5 (−4.1 to 3.1)	−0.8 (−6.1 to 4.5)	0.01 (0.9385)

^‡^Adjusted for SES and intervention arm.

## Discussion

In this study, examining linkage to care among a rural population in South Africa, we found that linkage to care was high (88.4%) within 3 months of HIV diagnosis irrespective of built environment conditions. Our linkage to care findings were higher than results from prior South Africa linkage to care studies. A community cross-sectional survey in rural KwaZulu Natal, South Africa found linkage to care was 71.0% (95% CI: 68.6 to 73.4).^39^ In addition, findings from a community-based study in Western Cape, South Africa indicate linkage to care was 63.1%.^14^ Lastly, a national representative survey conducted in South Africa reported that the proportion of linkage to care in 2016 was 21.9% (95% CI: 14.9 to 31.0).^40^ However, the definitions of linkage to care varied throughout the studies listed above.

We found a significant association between residential density and HIV linkage to care at both 3 months and 12 months post-diagnosis. Those with better residential density conditions were more likely to link to care than those who did not have better residential density conditions. Higher residential density typically means there are more destinations such as health care services and public transport stops nearby,^41^ which might be make it easier to access care. Previous studies have found an association between neighborhood of residence and utilization of healthcare.^42–47^ For instance, a study among American rural communities in the Deep South found that poor built environment was a barrier to HIV care utilization, in particular, transportation or distance to care.^43^ Furthermore, we did not find a significant association between land-use mix and aesthetics with linkage to care. In our study we assumed residents utilized easily accessible health facilities such as those nearby or in the same village. However, there is a possibility that residents were reluctant to seek HIV care in health facilities located in their village due to HIV stigma or discriminate against people living with HIV. The influence of stigma or discrimination might outweigh the impact of built environment. Research has shown stigma and discrimination in health facilities as a challenge to confidentially.^28^

The implementation of the universal test and treat policy seemed to have modified the association between land-use mix and HIV linkage within 12 months of diagnosis; similarly, an East African study found increases in LTC in the context of UTT.^8^ Ideally, with the implementation of UTT, individuals can initiate ART on the day of HIV diagnosis, reducing the need for multiple health facility visits and thus reducing the impact of built environment on future visits required to ensure linkage. Moreover, there was no evidence to suggest presence of effect measure modification for land-use within 3 months of diagnosis, and for residential density and aesthetics at both 3 and 12 months.

As a limitation, the randomly selected coordinates might not have been an accurate representation of built environment conditions for all village residents. Additionally, the Neighborhood Environment Walkability Scale was designed to assess neighborhood built environment specifically for physical activity; it might be an inappropriate tool to use for outcomes unrelated to physical activity.^35^ Although NEWS consists of features of urban areas (e.g. the housing patterns), it is a tool suitable for capturing built environment in rural settings as well. NEWS was adapted to assess built environment across South Africa, including both in urban and rural areas. Also, in our study, we were limited to using subjective measures to capture built environment. Another limitation is that we did not capture those who sought care at private facilities, however, only a small amount of AHDSS residents utilize private healthcare facilities.^48,49^ Lastly, due to substantial labor migration in the AHDSS area, patients classified as not linked to care or out of care may be in care at destinations outside the AHDSS.^31^ As a strength, three raters assessed built environment conditions at each location. Additionally, our study used a comprehensive definition of HIV LTC. Most studies consider HIV LTC solely based on 1) evidence of CD4/viral test,^50–52^ or 2) on attendance of first HIV healthcare apppointment.^53^ LTC definitions exclusively based on CD4 testing might not be a good indicator in the context of UTT because ART initiation is now independent of CD4 count.

## Conclusion

This analysis provides an examination of the relationship between built environment features and HIV linkage to care in rural South Africa. Our results suggest that high residential density is associated with HIV linkage to care. Also, findings indicate, high land-use mix had a protective effect on linkage to care within 12 months after the UTT policy was introduced, but that other environment conditions (density and aesthetics) were not significantly associated with HIV linkage to care. Further research is needed to understand how location affects linkage to care, and appropriate measures are needed for rural communities to capture various aspects of built environment and tools that can be utilized for a range of health outcomes. Research on neighborhood features might provide a possible pathway to increase uptake of HIV linkage to care in rural communities.
